# Supplementary material for: Proteome Analysis of Aflibercept Intervention in Experimental Central Retinal Vein Occlusion
Source: Molecules. 2022 May 24;27(11):3360. doi: 10.3390/molecules27113360 (PMC9182497; doi:10.3390/molecules27113360)
Supplement: Supplementary file 1 [file molecules-27-03360-s001.zip › Figure S1 - Additional immunohistochemistry confirming the reproducibility of the CRVO model.pdf]

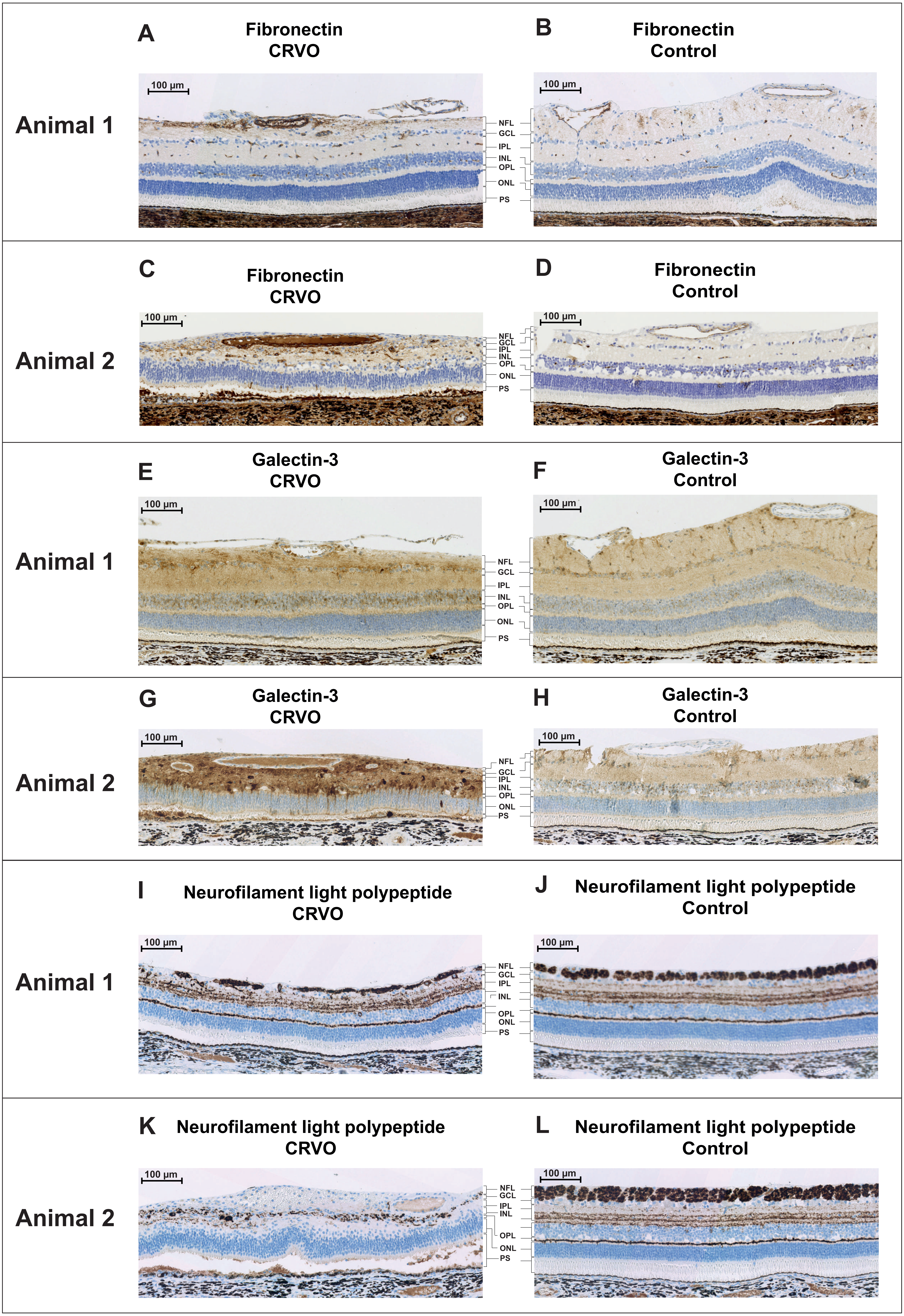

Animal 1
A
Fibronectin
CRVO
100 µm
B
Fibronectin
Control
100 µm
NFL
GCL
IPL
INL
OPL
ONL
PS
Animal 2
C
Fibronectin
CRVO
100 µm
D
Fibronectin
Control
100 µm
NFL
GCL
IPL
INL
OPL
ONL
PS
Animal 1
E
Galectin-3
CRVO
100 µm
F
Galectin-3
Control
100 µm
NFL
GCL
IPL
INL
OPL
ONL
PS
Animal 2
G
Galectin-3
CRVO
100 µm
H
Galectin-3
Control
100 µm
NFL
GCL
IPL
INL
OPL
ONL
PS
Animal 1
I
Neurofilament light polypeptide
CRVO
100 µm
J
Neurofilament light polypeptide
Control
100 µm
NFL
GCL
IPL
INL
OPL
ONL
PS
Animal 2
K
Neurofilament light polypeptide
CRVO
100 µm
L
Neurofilament light polypeptide
Control
100 µm
NFL
GCL
IPL
INL
OPL
ONL
PS
